# Kazuto Kato: the ethics of editing humanity

**DOI:** 10.2471/BLT.21.030921

**Published:** 2021-09-01

## Abstract

Gary Humphreys talks to Kazuto Kato about the ethical and societal challenges posed by biotechnologies that allow for the editing of the human genome.

*Q: You started out working in developmental biology. How did you become interested in the field of ethics and governance of biomedicine?*

A: As a student, I always had a broad interest in the societal issues related to biological science and its application and was actually a member of a student club that focused on such issues. I was also encouraged to look at issues outside the laboratory by my uncle, Professor Shuzo Nishimura, a pioneer in the field of health economics in Japan. Of course, I also had nine years at Kyoto University and four years at the University of Cambridge with Sir John Gurdon studying biology, with a focus on stem cell biology and genomics. This background has helped me navigate what is sometimes complex terrain.

*Q: Given that complexity, can you start by giving our readers a simple definition of genomics and gene editing?*

A: Genomics is the interdisciplinary field that focuses on the genome which is an organism's complete set of DNA. Gene editing falls within that field and is a type of genetic engineering.

*Q: The debate around genome editing has become focused on the potential applications of CRISPR-Cas9 (clustered regularly interspaced short palindromic repeats; Cas9 nuclease). Why is this?*

A: The genomics debate can be dated back to the cloning breakthroughs that have come to be identified with Dolly the sheep and breakthroughs made since, notably the development of induced pluripotent stem (iPS) cells derived from somatic (non-heritable) cells that was pioneered by Professor Shinya Yamanaka in Kyoto. But CRISPR-Cas9 has received a lot of attention because it is so powerful, cheap, and easy to use. It is also widely available. The unprecedented ‘democratization’ of what is an extremely powerful technology presents us with some profound ethical challenges.

*Q: Can you explain in simple terms how CRISPR-Cas9 works?*

A: The CRISPR gene repeats are found in bacterial DNA and are used by bacteria to identify viruses. Cas9 is an enzyme that can cut DNA. So, basically, bacteria use CRISPR to spot ‘bad’ viruses then send the Cas9 enzyme to cut them up. Professors Emmanuelle Charpentier and Jennifer Doudna worked out how to re-programme these genetic scissors to cut any DNA molecule at a predetermined site, making them powerful gene editing tools.

*Q: What are these tools being used for?*

A: To develop new treatments for genetic disorders as well as to prevent them, to develop new diagnostics and explore new approaches to treating infertility, all of which could have a huge impact on public health. On the treatment front, a good example is the use of somatic human genome editing to combat certain blood disorders, notable among them sickle cell anaemia. Human trials have already started for such treatments. As exciting as all that is, there are still questions about potential benefits and risks, and gaps in our understanding regarding important issues, including off-target effects where Cas9 cuts the wrong genomic sites, something that can lead to genetic mutations. But there are of course broader ethical and societal concerns, including, for example, ensuring equitable access to such interventions.

“CRISPR-Cas9 could bring a great deal of harm…”

*Q: To date, gene therapies have been extremely expensive, with some costing millions of dollars. How do we make them more affordable?*

A: Clearly, this is a major challenge, and it will require concerted efforts on several fronts, including improving transparency on research, development and manufacturing costs, and addressing different market failures. One concern is how to manage intellectual property. To explore the potential for the adoption of ethical licensing agreements it may be helpful for WHO to convene meetings of those holding or applying for patents relevant to human genome editing, as well as industry bodies and international organizations, such as the World Intellectual Property Organization and the World Trade Organization. Those involved in developing relevant patent pools should also be involved in the discussions.

*Q: Considerable attention has focused on heritable germline editing in debates regarding human genome editing. Why is this a central concern?*

A: First, it is important to note that not all research on germline cells involves heritable modifications. This is true for example of in vitro studies on early embryos and gametes (sperm and ova). However, some research does involve the editing of embryos that are then used to establish pregnancies and create individuals who could pass on the edit to their offspring. This includes research on the application of new biotechnology combining iPS cells and CRISPR-Cas9.

*Q: Can you say more about that?*

A: So far scientists have only produced gametes from iPS in mice, and produced offspring from those cells, but if they succeed in applying the technology to human cells, it will be possible in theory to edit iPS cells using CRISPR-Cas9, select the most successful edits – i.e. those without off-target edits or other disturbance to the genome – then produce multiple iPS-derived gametes and use those gametes to create new embryos. This will make the whole process more effective and possibly safer. Notwithstanding such developments, so-called heritable human genome editing is not currently safe and raises considerable ethical concerns, since its potential impact is felt not just by the immediate recipients, but by subsequent generations. In recognition of these issues, the WHO expert advisory committee recommended that there be no work on such applications at this moment. The Director-General also stated that it would be irresponsible for anyone to proceed with clinical applications of this kind of editing at this time. Unfortunately, we have already seen examples of rogue clinics planning to use heritable modification to enhance or otherwise ‘improve’ individuals.

*Q: You mention ‘rogue clinics’. Can you explain what is meant by that term?*

A: Clinics or scientists offering services that have no scientific basis or which have not gone through the trials required to establish safety and efficacy. For example, currently a number of clinics are offering so-called regenerative stem cell interventions using stem cells and stem cell-derived components, supplying a worldwide, direct-to-consumer market. In some instances, services have been offered by individuals, as in the case of the researcher who, with the intention of preventing HIV (human immunodeficiency virus) infection, implanted genetically modified human embryos, resulting in the birth of twin babies with a heritable germline modification.

“We need dialogue and transparency now more than ever.”

*Q: Given that the technology is so accessible, cheap and easy to use, what are the prospects for regulating its use?*

A: It is going to be extremely challenging and will require a global, collaborative effort pulling on different regulatory and governance levers. Again, WHO can play a key role, both in developing a shared vision and exploring opportunities for collaborative engagement. Specifically, we are going to need agreed-upon standards and oversight mechanisms developed in consultation with national regulators and other key stakeholders including patient groups and civil society organizations. Fortunately, there are already laws, regulations and guidelines developed for medical products by trusted regulatory agencies that have relevance for CRISPR-Cas9-related products and applications. The expert advisory committee is also calling for the development of a set of international standards for clinical trials involving human genome editing and the establishment of a human genome editing clinical trial registry. To be registered, clinical trials using somatic human genome editing technologies will have to be reviewed and approved by the appropriate research ethics committee.

*Q: But how do you regulate rogue actors?*

A: First, I think it is important to be extremely clear what the red lines are and to establish some system of sanctions, just as we have with nuclear technologies. However, it is also important to recognize the limits of regulation. It is for this reason that the expert advisory committee emphasized the importance of collaborative approaches ensuring transparency and concluded that we should do all we can to make sure that inappropriate use is reported. Notable in this regard, is the committee’s call for the establishment of an accessible mechanism for confidential reporting of concerns about possibly unethical and unsafe human genome editing activities. It has been proposed that WHO’s Science Division lead the effort to create a multisector collaboration to develop such a mechanism.

*Q: How optimistic are you that such a whistle-blowing mechanism will be sufficient?*

A: Personally, and as a matter of principal, I refuse to accept that we will not be able to control this technology, but I also believe we need to be clear-eyed about the challenges we face. Without appropriate governance, CRISPR-Cas9 could bring a great deal of harm into human society. In light of this, I believe that regulators need to consider establishing international agreements and possibly an enforceable international treaty governing limits of emerging technologies including but not limited to CRISPR-Cas9 use. It is of the utmost importance that we recognize that this is a very powerful technology, comparable in some ways to nuclear fission and fusion technologies for which we have relatively strong treaties, despite the spotty history of their implementation.

*Q: You make the comparison with nuclear technologies, but, given its accessibility, isn’t the threat posed by this technology different and, notwithstanding the prospect of nuclear war, perhaps even greater?*

A: In a way yes. But there are still points of comparison. Perhaps as a Japanese citizen, I am particularly sensitive to those parallels. I am reminded of Barack Obama’s address at Hiroshima in 2016 when he talked about the power of science and the effort required to make the best use of it. He said the scientific revolution requires a moral revolution. I don’t think the challenge has ever been more clearly stated.

